# Cytoimmunological Profile of Lower Airways in Post-COVID-19 Syndrome (PCS): Predictive Value of Bronchoalveolar Lavage

**DOI:** 10.3390/jcm14103361

**Published:** 2025-05-12

**Authors:** Justyna Dolna-Michno, Piotr Kopiński, Grzegorz Przybylski, Ewa Wypasek, Magdalena Szymańska, Ewelina Wędrowska, Klaudia Mikołajczyk, Tomasz Senderek, Maciej Gagat

**Affiliations:** 1 Department of Molecular Biology, John Paul II Hospital, 31-202 Kraków, Poland; mpkopins@cm.umk.pl (P.K.); ewa.wypasek@wp.pl (E.W.); szyman.mag@gmail.com (M.S.); 2 Department of Physiology and Pathophysiology, Medical College, Andrzej Frycz Modrzewski Krakow University, 31-216 Kraków, Poland; tomeksenderek@gmail.com; 3 Department of Lung Diseases, Cancer and Tuberculosis, Collegium Medicum, Nicolaus Copernicus University, 85-067 Bydgoszcz, Poland; gprzybylski@cm.umk.pl (G.P.); e.wedrowska@cm.umk.pl (E.W.); 4 Department of Histology and Embryology, Collegium Medicum, Nicolaus Copernicus University, 85-067 Bydgoszcz, Poland; klaudia.mikolajczyk@cm.umk.pl (K.M.); mgagat@cm.umk.pl (M.G.)

**Keywords:** bronchoalveolar lavage, interstitial lung disease, lung lymphocytes, lung neutrophils, post-COVID syndrome, T exhausted cells, Th17 cells

## Abstract

**Background**: It has yet to be determined whether the immunocytological profile of the bronchoalveolar lavage (BAL) in respiratory post-COVID syndrome (PCS) reflects the risk of persistent interstitial lung disease (ILD), including pulmonary fibrosis. In this study, we aimed to assess the prognostic value of the BAL cytoimmunologic profile in PCS-related ILD. **Materials and Methods**: We enrolled 58 non-smoking patients with a history of COVID-19 and new-onset ILD, divided into PCS remission and PCS persistence groups based on clinical data, including repeated computed tomography and pulmonary function tests. We phenotyped BAL major T cell subsets, immune checkpoints (including programmed cell death-1, PD1), and markers of Th1/Th2/Th17 polarization. **Results**: The PCS groups compared to the control showed increased total cell, lymphocyte, and neutrophil counts and a high BAL neutrophil:lymphocyte ratio (NLR). PCS persistence compared to the controls presented an increased neutrophil count (26 [17–36] vs. 2.6 [1.9–5.4] 10^3^/mL, median [Q1–Q3], *p* < 0.001) and percentage, BAL NLR (0.77 [0.26–1.63] vs. 0.21 [0.17–0.31], *p* < 0.0001), CD8+PD1+ cell percentage (43.5 [34–60.5] vs. 24.5 [22–44]%, *p* = 0.045), and a decreased CD4:CD8 ratio. A high percentage of CD4+CD196+CD183 cells (relevant to Th17 activity, 6.2 [2.0–9.4] vs. 1.2 [0.7–2.7]%, *p* = 0.02) and increased BAL supernatant elevated IL-8 levels (62.5 [16–243] vs. 10.9 [3.44–32] pg/mL, *p* = 0.002) were found in the PCS persistence vs. control groups. In the total PCS group, predicted values of Vital Capacity (VC) [16–243] and Diffusing Lung Capacity for CO (DLCO) correlated negatively with BAL NLR; VC correlated negatively with BAL CD8+PD1+; and DLCO correlated positively with the CD4:CD8 ratio. **Conclusions**: Worse prognosis in PCS is associated with higher BAL NLR, BAL neutrophilia, an elevated percentage of CD8+PD1+ lymphocytes, and a decline in the CD4:CD8 ratio. Th17 cells and IL-8 participate in lung PCS persistence.

## 1. Introduction

The COVID-19 pandemic, caused by infection with the SARS-CoV-2 virus, was declared a pandemic by the World Health Organization (WHO) on 11 March 2020. In the three years since the pandemic began, virus variants have caused 776.8 million confirmed cases and over 7 million associated deaths worldwide as of 10 November 2024 [1].

While most COVID-19 patients recover and have a low mortality rate [2], the chronic features of SARS-COV-2 infection have become increasingly apparent. These common, long-lasting symptoms affect 50–70% of hospitalized acute COVID-19 cases and 10–30% of non-hospitalized cases (with a total of 5–50%, depending on the study) and have been given alternative names and slightly different definitions [3]. The current study used the term post-COVID-19 syndrome (PCS); other commonly used terms include long COVID, post-acute COVID-19 syndrome (PACS), post-acute sequelae of COVID-19, and chronic COVID syndrome [4,5,6].

The WHO defines PCS as a syndrome that may occur either as a continuation of the acute illness or as a consequence of a previous infection, with the appearance of new symptoms that were not previously present [5]. Symptoms may last for weeks, months, or years (at least two weeks) and may fluctuate or relapse over time. The diagnosis of PCS requires that clinical symptoms were not present prior to infection, persisted for at least two months, cannot be explained by an alternative diagnosis, and occurred at least 12 weeks after the onset of the acute illness, with patients experiencing significant impairment in daily functioning [7,8].

PCS lesions primarily include respiratory, cardiovascular, coagulation, and mental disorders [9]. Respiratory changes are the most common; at the beginning of the pandemic, SARS-COV-2 infection was reported to cause permanent respiratory impairment in patients after hospital discharge [10]. They are often the result of interstitial lung disease (ILD) diagnosed in patients with PCS, also referred to as persistent ILD COVID-19 [11]. Typical respiratory complaints associated with PCS include fatigue, decreased exercise tolerance, coughing, and dyspnea. Clinical findings include abnormal CT images of the lungs and impaired pulmonary function tests [7].

The pathogenesis of PCS remains unclear. Research has focused on two key mechanisms: the survival of viral antigens in the lower respiratory tract with an ineffective chronic inflammatory response [12] or, conversely, the complete elimination of the virus with local autoimmunity [13]. The dysregulation of the immune system, particularly of T cells, including changes in the reciprocal proportions of effector Th1, Th2, and Th17 cells, is possible [14]. Changes in the expression of immune checkpoints, i.e., molecules expressed on lymphocytes that regulate the intensity of the immune response and ultimately cause apoptotic lymphocyte death, have been detected [15]. The significant increase in the percentage of CD8+PD1+ cells found in PCS is thought to indicate a down-regulation of the antiviral potential of cytotoxic T cells (exhaustion phenomenon) [16].

In addition to lymphocytes, neutrophils are important cells in the lower airway inflammatory response in patients with PCS [17]. Their recruitment to the lung interstitium may depend on Th17 effector lymphocytes; however, to our knowledge, this possibility has not been tested.

Examination of bronchoalveolar lavage (BAL) fluid has been shown to have diagnostic and clinical relevance in acute SARS-COV-2 infection [18]. In contrast, less is known about the cytological and immunological profiles of BAL in the PCS group [19].

In this study, we aimed to evaluate the immunocytological profile of PCS in the lower respiratory tract, including the subgroup with persistent changes without current remission. Therefore, we tried to identify (1) BAL changes characteristic of PCS and (2) BAL abnormalities responsible for worse prognosis in PCS.

## 2. Materials and Methods

### 2.1. Patients

We enrolled 58 patients from the Department of Pulmonary Diseases, John Paul II Hospital, Krakow, and the Department of Pulmonary Diseases, Cancer, and Tuberculosis, Nicolaus Copernicus University, Bydgoszcz, who were initially suspected of having new-onset ILD. These patients had BAL samples taken between August 2020 and May 2023 as part of a diagnostic work-up and were ultimately diagnosed with PCS. The diagnostic procedure followed the European Respiratory Society guidelines [20].

All of the subjects enrolled had a history of acute COVID-19 infection confirmed by PCR or antibody testing in a medical facility or at home. They were enrolled regardless of the severity of the infection or the pandemic wave.

We excluded patients: (1) those with a history of previous interstitial lung disease; (2) those treated with systemic corticosteroids, methotrexate, amiodarone, biologic drugs, or other drugs known to be a potential cause of interstitial lung pathology; (3) those with New York Heart Association class IV heart failure, respiratory failure, or acute coronary syndromes; (4) those with evidence of a respiratory infection other than SARS-COV-2 (such as tuberculosis, influenza, RSV virus, mycoplasma, bacterial infections, etc., although the presence of physiological bacterial flora was considered acceptable) [2,3,20,21,22].

Smokers were not analyzed in the study because the number of PCS smokers with BAL examination was insignificant and there were too few smokers in the control group for statistical analysis.

All enrolled patients underwent pulmonary function testing prior to bronchoscopy. They all reported respiratory symptoms and abnormal lung computed tomography (CT) scans showing interstitial lung lesions. The following were considered eligible for extended diagnosis: on chest CT, ground-glass opacities, consolidations, linear and reticular lesions, honeycombing, traction bronchiectasis, interlobular septa thickening, and reduced lung volumes, including vital capacity (VC). The distinction between fibrotic and non-fibrotic symptoms in PCS was made in this study using the Fleischner Society dictionary [23]. Traction bronchiectasis, honeycomb lesions on CT, and VC volume loss were considered fibrotic changes. In fact, small residual fibrotic changes may occur as a direct consequence of COVID-19-related ARDS and are found in PCS remission; VC and diffusing lung capacity for CO (DLCO) remain normal in these patients [3].

Patients were assessed for known risk factors for PCS, including age, sex, comorbidities (arterial hypertension, asthma, chronic obstructive pulmonary disease (COPD), diabetes, and coronary artery disease), vaccination status, and severity of acute COVID-19 (subdivided into mild, moderate, severe, and critical). Severe (usually pneumonia with hospitalization) and critical (usually ARDS) acute COVID-19 are considered risk factors for PCS [3,23,24].

The time of BAL examination was at least 12 weeks (3 months) from the end of acute SARS-CoV-2 infection [25].

Patients underwent follow-up CT lung examinations and functional tests at least 12 months after the BAL and baseline CT lung examinations; this was carried out as suggested by other authors [7,25,26]. Patients were divided into two groups according to the dynamics of interstitial lung lesions, ventilation, and diffusion disorders:

(1) Interstitial lesions with complete or partial resolution by 12 months after the first examination with predictive values of VC and DLCO greater than 80%; these patients were defined as PCS remission (n = 33).
(2) Persistent interstitial lesions (defined as both non-fibrotic and fibrotic) observed at 12 months from baseline with associated ventilatory compromise (with VC and/or DLCO predicted values less than 80%); patients were defined as having PCS persistence (n = 25).

Exceptionally, patients with small fibrotic lesions, assessed as residua following acute illness (especially after COVID-related ARDS), that showed no enlargement and no ventilatory compromise were classified in the PCS remission subgroup [3].

We did not identify the variants of the SARS-COV-2 virus in individual patients.

The treatment strategy for PCS patients has included combined breathing and light exercise rehabilitation and inhaled steroids for symptomatic treatment [27]. Of note, no drug has been shown to be effective in relieving PCS symptoms in controlled or large cohort studies; there is no credible evidence to support the routine use of systemic glucocorticosteroids in PCS-ILD. Antifibrotic therapies, such as pirfenidone and nintedanib, may be effective in slowing down pulmonary fibrosis [28]. Pirfenidone was administered in three study patients with persistent PCS; the therapeutic decision was made after the follow-up period. All unvaccinated patients were advised to follow the standard SARS-CoV-2 vaccination schedule [29].

The control group consisted of 11 non-smoking subjects with no lung pathology, no symptoms of acute or chronic lung disease, and normal lung function tests, chest X-rays, and chest CT scans. They were not treated with corticosteroids or any other medication known to be a potential cause of ILD (see above).

Informed consent was obtained from all enrolled subjects (Bioethics Committee of Nicolaus Copernicus University, approval No. KB299/2022 of the day 17 May 2022, valid until 31 December 2023).

### 2.2. Bronchoalveolar Lavage (BAL)

BAL was performed as a routine diagnostic procedure according to European Respiratory Society (ERS) guidelines [15,22]. BAL fluid fractions were collected by gentle aspiration, combined, filtered, and immediately transported to laboratories. Fluid recovery was calculated as a percentage of the instilled volume. Total cell count, cell viability (trypan blue exclusion test), and differential white blood cell count [20] were calculated.

BAL samples with fluid recovery <30% of the instilled volume and epithelial cell contamination >3% of BAL cells were excluded from further analysis due to poor technical quality [22].

### 2.3. BAL Lymphocyte Phenotyping and Flow Cytometry (FC)

All BAL samples met the exact criteria for cytometric material collection and analysis [15]. Five-color direct typing was used (flow cytometer BD FACSCantoTM II, BD Immunocytometry Systems, San Jose, CA, USA). BAL samples containing 50 μL of cell suspension (2–10 × 10^6^ cells/mL) were incubated with saturating amounts of mouse fluorochrome-conjugated monoclonal antibodies to human surface markers: CD3, CD4, CD8, CD16+56, CD19, HLA-DR, CD45, CD152 (CTLA4), CD183 (CXCR3), CD194 (CCR4), CD196 (CCR6), CD274 (PDL1), and CD279 (PD1); monoclonal antibodies were purchased from Becton-Dickinson Biosciences, San Jose, CA, USA and Biolegend, San Diego, CA, USA for 30 min in the dark, washed in PBS (phosphate-buffered saline), and then resuspended in 500 μL PBS containing 1% formaldehyde. The internal control consisted of samples stained with the negative isotype control [20], with between 10,000 and 100,000 cells collected per sample. Alveolar macrophages, alveolar lymphocytes (AL), and BAL granulocytes were graded according to cell granularity (side scatter, SSC) and intensity of CD45 staining. The AL phenotype was obtained by dot plot quadrant analysis of the corresponding fluorescent channels [18]. BAL lymphocyte phenotyping included: (1) major subset typing; (2) assessment of Th effector cell subpopulations (Th1-like cells such as CD4+CD183+CD196−; Th2-like cells such as CD4+CD194+CD183−CD196−; and Th17-like cells such as CD4+CD183−CD196+) [30]; and (3) immune checkpoints, ICP, expression study on Th and Tc cells, including the percentage of CD4+PD1+ and CD8+PD1+ cells, i.e., exhausted T cells [31].

The set of monoclonal antibodies used in the study is presented in the Supplementary Material, Table S1. The representative dot plots of the flow cytometry analysis are shown in the Supplementary Material, Figure S1.

### 2.4. Enzyme-Linked Immunosorbent Assay (ELISA): BAL Cytokines/Chemokines

The concentration of cytokines (IL-5, IL-7, IL-8, IL-10, IL-15, IL-17, IFNγ, and TGFβ1) in BAL supernatants was determined by enzyme-linked immunosorbent assay (ELISA) kits (R&D Systems, Quantikine cat. no. IL-5: D5000B, IL-7: HS750; IL-8: D8000, IL-10: D1000B, IL-15: D1500, IL-17: HS170, IFNγ: HSDIFO, TGFβ1: DB100C, Minneapolis, MN, USA) according to the manufacturer’s recommendations. Optical density was measured at 450 nm or 490 nm using an ELx808 spectrophotometric reader (Biotek Instruments Inc., Winooski, VT, USA). The results were expressed in pg/mL.

## 3. Statistics

Statistical analyses were performed using STATISTICA version 12.5 (Stat Soft STATISTICA^TM^, Kraków, Poland).

Patient age and lung function data are expressed as mean ± SD (standard deviation); cytokine concentrations in the BAL supernatant, AL staining results, and cytology data are presented as median, minimum-maximum range, and interquartile range [Q1–Q3] due to the non-parametric distribution of values [15]. The Mann-Whitney U test was used to compare groups (PCS subgroups with each other and with the control group). Correlations between the two random variables were tested using Spearman’s rank correlation coefficient r. *p*-values less than 0.05 were considered statistically significant.

## 4. Results

The baseline characteristics of patients with PCS are presented in Table 1.

Patients in the PCS persistence subgroup were more likely to be male and older (statistical trend, *p* = 0.09) than those in PCS remission. PCS patients had significantly lower DLCO predicted values, especially when the persistence subgroup was considered. Baseline data were supplemented with clinical findings known to be risk factors for acute COVID-19, such as C-reactive protein and peripheral blood neutrophil:lymphocyte ratio (Table 1).

PCS was characterized by significantly increased BAL total cell counts, as well as elevated lymphocyte, T, Th, Tc, and NK cell counts, and a higher percentage of neutrophils compared to the controls. When the neutrophil results were converted to total cell counts, the increase was 17.6 [8.3–41] in PCS vs. 2.6 [1.9–5.4] 10^3^/mL in controls (*p* < 0.001).

The differences between the subgroup with persistent PCS and controls mirrored those between all PCS and the controls. Of note, the neutrophil count was 26 [17–36] in PCS persistence vs. 11.2 [5.9–27] in PCS remission, *p* = 0.012, and 2.6 [1.9–5.4] 10^3^/mL vs. controls, *p* < 0.001.

**Figure 1:**
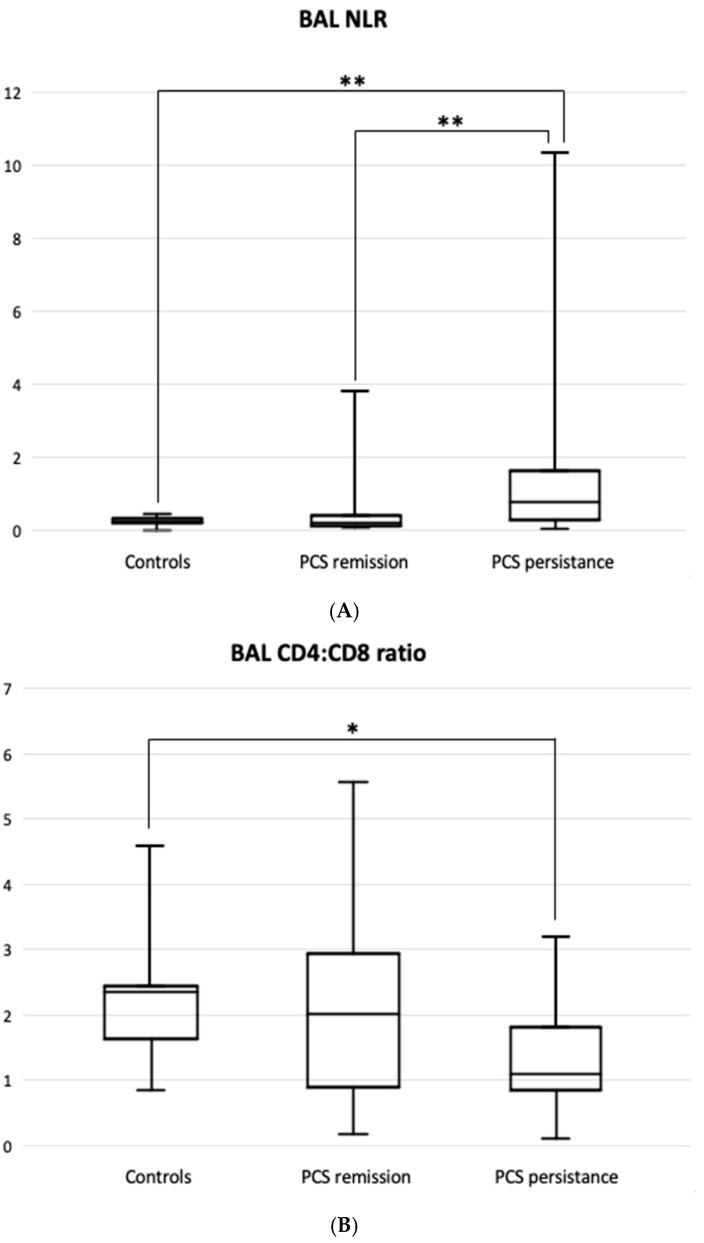
BAL neutrophil:lymphocyte ratio, NLR (**A**), and BAL CD4:CD8 ratio (**B**) in patients with PCS resistance compared to PCS remission and healthy controls. Horizontal middle lines express medians. Boxes express interquartile range (Q1–Q3). Whiskers indicate minimum-maximum range. * *p* < 0.05, ** *p* < 0.01.

The percentage of BAL lymphocytes was significantly higher in the PCS remission group than in the control group, but not in the PCS resistance group (Table 2). In summary, the BAL neutrophil:lymphocyte ratio best reflected the difference between the PCS resistance group and the PCS remission group (0.77 [0.26–1.63] vs. 0.23 [0.12–0.40], *p* < 0.01) as well as the controls (vs. 0.21 [0.17–0.31], Figure 1A).

The BAL CD4:CD8 ratio was significantly lower in patients with PCS resistance than in the controls, 1.09 [0.85–1.81] vs. 1.98 [1.65–2.41], *p* = 0.016 (Figure 1B).

**Figure 2:**
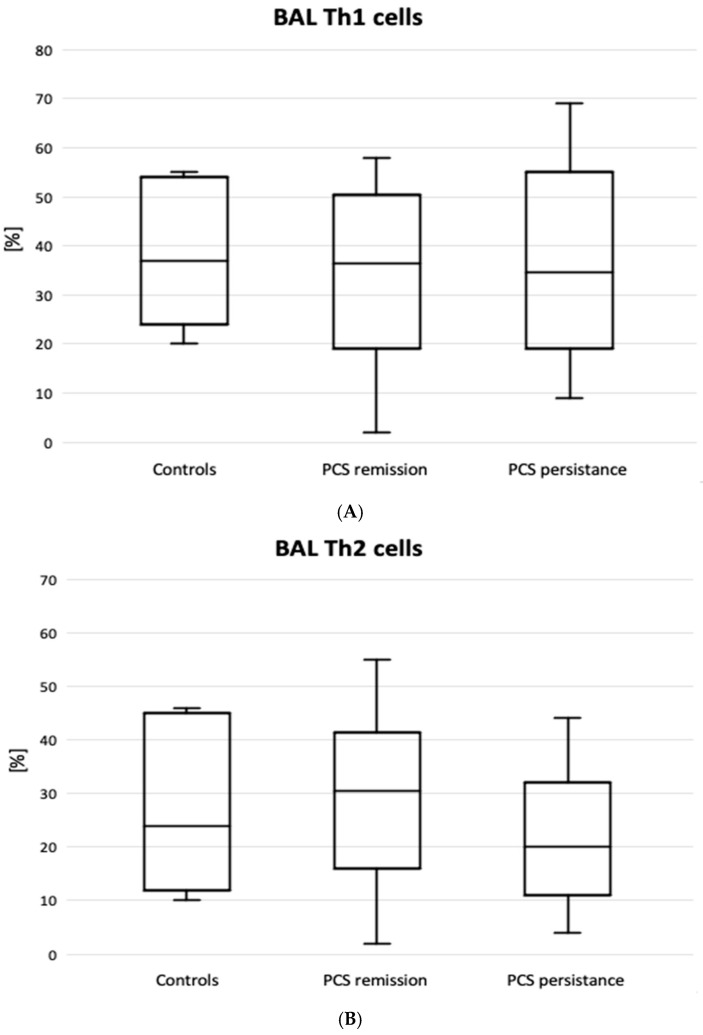

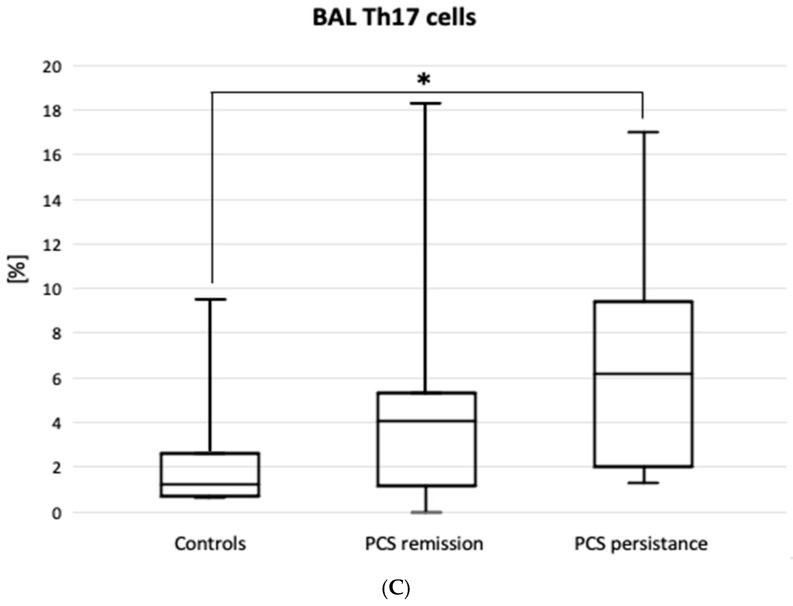
T helper 1, Th1 (**A**), T helper 2, Th2 (**B**), and T helper 17, Th17 (**C**) in patients with PCS resistance compared to PCS remission and healthy controls. Horizontal medians express median values. Boxes express interquartile range (Q1–Q3). Whiskers indicate the minimum-maximum range. * *p* < 0.05 (for PCS resistance vs. controls).

Figure 2 shows the results of lymphocyte typing for Th1, Th2, and Th17. There was an increase in the percentage of Th lymphocytes with Th17 cell-specific markers (CD4+CD183-CD194+) in the PCS persistence group compared to the control group (6.2 [2.0–9.4] vs. 1.2 [0.7–2.7]%, *p* = 0.02). There were no significant differences between the PCS subgroups and the control group in the expression of Th1, Th2 (Figure 2), PDL1, and CTLA4 markers. However, there was a significantly higher percentage of CD8+PD1+ cells in the BAL of patients with PCS persistence compared to the controls (Table 3).

**Figure 3:**
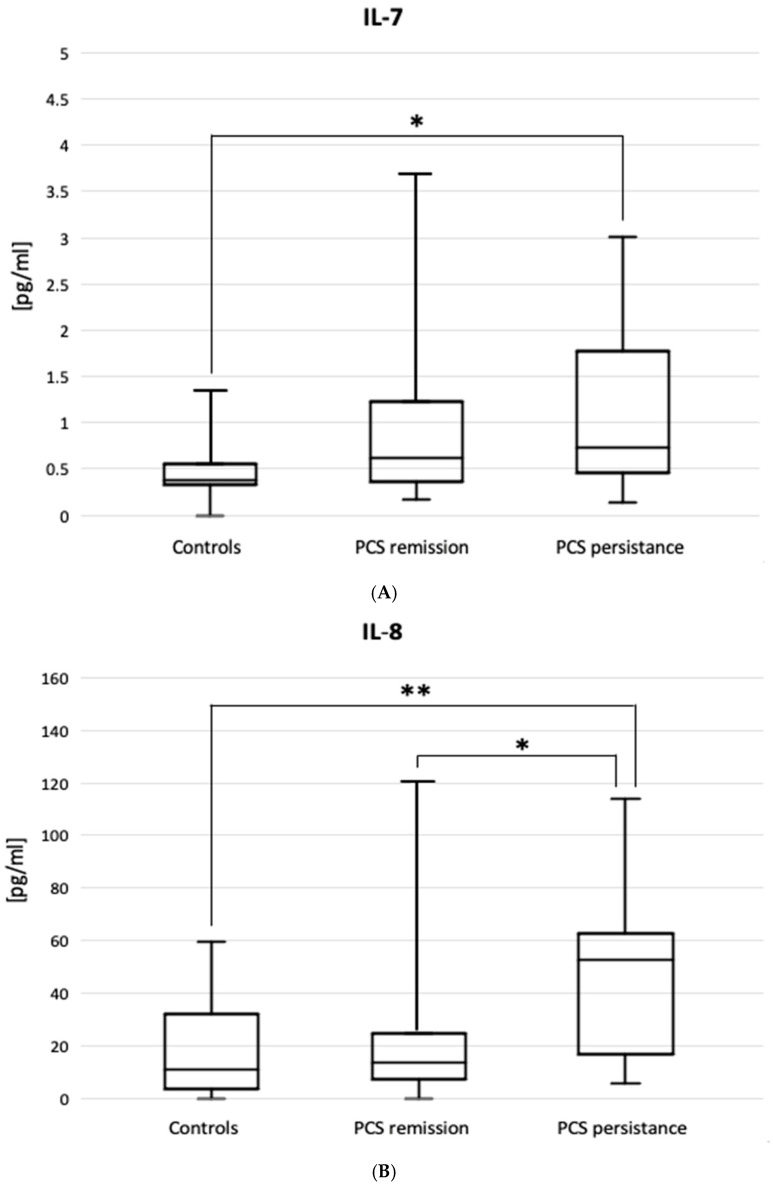

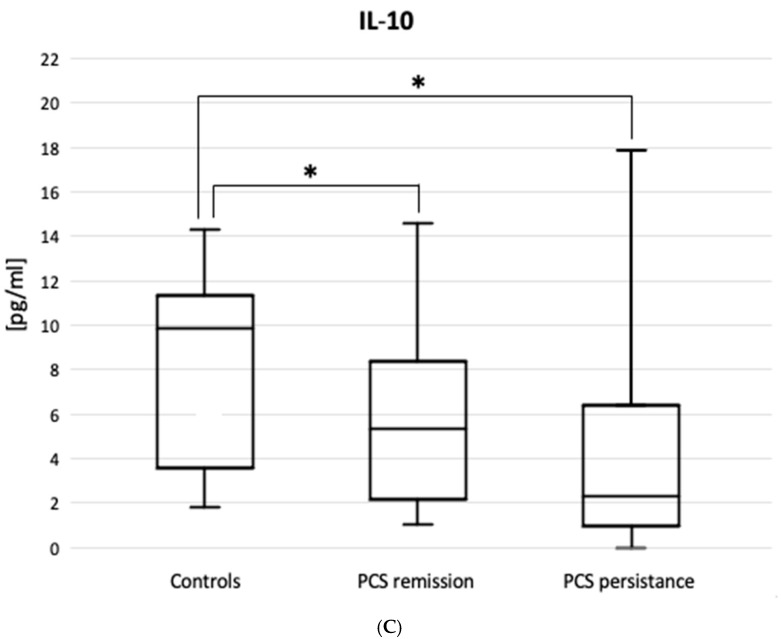
Interleukin-7, IL-7, (**A**), interleukin-8, IL-8 (**B**), and interleukin-10, IL-10 (**C**) in patients with PCS resistance compared to PCS remission and healthy controls. Horizontal medians express median values. Boxes express interquartile range (Q1–Q3). Whiskers indicate the minimum-maximum range. * *p* < 0.05, ** *p* < 0.01.

The ELIS results are shown in Figure 3. Patients with PSC persistence had higher BAL levels of IL-7 (0.70 [0.4–1.71] vs. 0.39 [0.34–0.56] pg/mL, *p* = 0.030) and IL-8 (62.5 [16–243] vs. 10.9 [3.44–32] pg/mL, *p* = 0.002), but lower BAL levels of IL-10 (2.58 [0.97–7.77] vs. 6.62 [1.79–11.3] pg/mL, *p* = 0.034) compared to the controls. The figure does not include the results of the IL-5, IL-15, IL-17, IFNγ, TGFβ1, and TNFα ELISAs, as no statistically significant differences were observed between the patient subgroups or between the subgroups and the control group.

The description of the correlations in the study material focused on the relationships associated with VC and DLCO, assuming that these two parameters are particularly important for assessing the prognostic value of BAL examination in PCS.

In patients with PCS persistence, VC was negatively correlated with BAL NLR (r = −0.43, *p* < 0.01) and BAL IL-17 level (r = −0.60, *p* < 0.05); DLCO was negatively correlated with BAL NLR (r = −0.41, *p* < 0.05). In addition, in the total PCS group, VC showed a negative correlation with total BAL NK cell count (r = −0.32, *p* < 0.05) and the percentage of BAL CD8+PD1+ cells (r = −0.60, *p* < 0.01). DLCO was negatively correlated with the percentage of BAL CD8+PD1+ cells (r = −0.41, *p* < 0.05) but positively correlated with the BAL CD4:CD8 ratio (r = +0.36, *p* < 0.05) and the number of weeks since the end of acute SARS-CoV-2 infection (r = +0.67, *p* < 0.05).

## 5. Discussion

ILD-type lesions have been observed almost since the early stages of the COVID-19 pandemic. As early as 2022, based on the data from the pandemic in northern Italy, Besutti et al. presented the results of a 5–7 month follow-up after COVID-19 pneumonia and reported residual non-fibrotic chest abnormalities in 37.5% of patients whilst fibrotic lesions were found in only 4.4% of cases [32].

To date, a significant number of PCS studies have focused on specific groups of COVID-19 patients, most of whom were hospitalised for acute infection, i.e., with severe or critical illness [3]. As a result, patients with mild/moderate SARS-CoV-2 infection are likely to have been underrepresented [33]. In this study, we proposed a different approach: instead of monitoring selected patients, we screened patients diagnosed with suspected ILD for evidence of PCS.

In the material presented in the study, up to 57% (33/58) of patients with ILD showed significant improvement and even complete remission. The prognosis for the remaining patients is uncertain, but it is worth noting that, according to a meta-analysis by de las Penas et al., only 30% of patients with PCS ILD develop lung lesions after two years of follow-up; these patients also recover gradually [2]. In addition, it is likely that the prevalence of overall PCS decreased during the last phase of the pandemic and decreased after vaccination [34]. However, in the current study, we found no significant differences in the percentage of unvaccinated patients between the PCS remission, PCS persistence, and control groups.

The risk of fibrotic changes in the lungs of patients following COVID-19 is low: according to Johnston et al., traction bronchiectases were rarely reported, and the presence of honeycombing was incidental (0.2%) [3]. Furthermore, Robey et al. analyzed a reduction in the incidence of fibrosis from 25 to 7% after a 4-month follow-up [35]. This trend of improvement is also reflected in the results of the current study: in the total PCS group, the predictive value of DLCO showed a positive correlation with the number of weeks since the onset of acute COVID-19.

In this context, the early concerns raised during the early phase of the pandemic—that SARS-CoV-2 infection might cause permanent respiratory disability in a significant proportion of patients after hospital discharge—have not been confirmed [10].

However, some recovered patients are at risk of developing PCS ILD with pulmonary fibrosis [36]. During the study, only two cases of progressive pulmonary fibrosis with respiratory failure were observed in our PCS persistence subgroup, both with multiple comorbidities. Thus, there remains a need to identify the subgroup with persistent ILD, to define the immunocytological changes typical of this group, and to monitor them for further development of pulmonary fibrosis [18].

Patient demographics and comorbidities prevalence in the study were consistent with the literature data. Known risk factors for persistent ILD after acute COVID-19 include age, being male, a history of severe/critical acute COVID-19, more than two comorbidities, and a predicted DLCO of less than 80% [9,32]. In the study, COPD was a comorbidity in only a few patients with PCS, but this was due to the exclusion of cigarette smokers.

Variants of the SARS-COV-2 virus in individual patients were not identified. It is worth noting that none of the pandemic waves (attributed to the different variants) were overrepresented in this study. However, current evidence suggests that the incidence of PCS was higher in those infected with the historical Wuhan variant than in those infected with the Alpha, Delta, or (especially) Omicron variants [37].

Bronchoalveolar lavage (BAL) has been proposed as a tool to increase the diagnostic sensitivity of COVID-19. BAL is a feasible procedure in confirmed or suspected cases of COVID-19, useful in guiding appropriate patient management, and has potential for research [18].

In this study, we demonstrated an increase in total BAL cells, lymphocytes, and neutrophils in PCS patients. BAL neutrophilia was more pronounced in the PCS persistence subgroup, whereas lymphocytosis was more pronounced in the PCS remission subgroup. This translates into differences in the neutrophil:lymphocyte ratio, which was significantly higher in the PCS persistence subgroup than in the PCS remission subgroup and controls.

To date, the BAL immunocytological profile in acute COVID-19 has been better studied. According to Gelarden et al., hospitalized COVID patients showed BAL lymphocytosis (predominance of T cells with variable, usually low CD4:CD8 ratios), the appearance of activated “plasmacytoid” lymphocytes, and an increased percentage of neutrophils [38]. The cited study concluded that a high percentage of BAL lymphocytes supports a worse prognosis in the course of acute SARS-CoV-2 infection. On the contrary, Liao et al. pointed out that neutrophilia and the depletion of tissue-resident alveolar macrophages were found in severe cases; mild cases were characterized by clonal expansion of CD8+ T cells [39]. According to Baron et al., BAL neutrophilia is typical of acute COVID; however, their study population was limited to patients with critical forms of the disease [40]. In summary, in the acute form of COVID-19, poor prognosis was associated with BAL neutrophilia and a high BAL NLR [41,42].

The current knowledge of ILD in post-COVID syndrome needs to be further expanded. The typical CT findings of NSIP or OP imply BAL neutrophilia and lymphocytosis [21,22]. In fact, an increased percentage of neutrophils and/or lymphocytes in the BAL occurs in patients with PCS ILD [25,43].

The comprehensive study by Vijayakumar et al. provided additional characterization of BAL material 3–6 months after discharge. They reported an increase in lymphocytes involving both T and B cells, with cytotoxic T cells associated with epithelial damage and airway disease, while B cells (and proinflammatory myeloid cells) correlated with the extent of lung CT abnormalities. The role of activated T cells (CD69+CD103+), the expression of Th1 cell markers (CXCR3), and the trend towards remission were highlighted [44].

The results of the present study are partially similar (this is especially true for the increase in the percentage of T-cell-dominant lymphocytes in PCS compared to the control group), although the increase in the percentage of Th1 cells was unchanged and a different activation marker (HLA-DR) for CD3 cells was used. A significant difference was the finding of an increase in the number of BAL neutrophils in the PCS persistence group. This demonstrated the prognostic role of BAL NLR. It should be noted that severe/critical COVID-19 was overrepresented in the cited study [44].

Although the present study found an increase in the total number of both CD4 and CD8 lymphocytes (with a significant increase in the PCS persistence group for CD8 only), there was a notable trend towards lower CD4:CD8 ratio values in patients with persistent ILD-type lesions. The same observations have been reported in the past in primary NSIP [45].

The predominance of CD8+PD1+ cells in the PCS persistence subgroup is consistent with the existing literature [46], but poses challenges in terms of interpretation. On one hand, our results indicate the presence of PCS-persistent T cells in the lower airways, characterized by a progressive loss of cytotoxic functions, impaired immune memory, and interleukin-10 dependence. On the other hand, these cells showed a significant negative correlation with the predictive values of VC and DLCO, which may indicate a direct involvement in interstitial lung damage. Concentrations of IL-10, the immunosuppressive cytokine [47], were significantly reduced in the PCS persistence group. In this situation, the hypothesis of local immune dysregulation [48], whereby the SARS-CoV-2 virus persists in the airways, leading to the exhaustion of cytotoxic T cells and a chronic inflammatory response, is plausible [12]. Other indirect evidence of immune dysregulation in a group of patients with persistent ILD is the increase in IL-7, an immune memory cytokine, highlighted in this study [47].

Notably, the vitro IL-10 blockade, which overrides the PD1 inhibitory signaling, can reverse T-cell exhaustion [46].

Another key finding of the present study was the demonstration of a significant increase in the percentage of cells with a Th17 helper lymphocyte phenotype. Together with Th1 cells, they are involved in the pathogenesis of acute COVID-19 [49]. Our findings, which also point to their involvement in PCS persistence, establish a pathophysiological chain linking Th17 lymphocytes, the IL-17 they release, the interleukin-8 dependent on the latter, and activated neutrophils.

To elaborate on this idea, Th17-driven interleukin-17 is a recognized factor of lung lesions in COVID-19, acting as a neutrophilic pro-inflammatory cytokine [47]. Our results showed no remarkable increase in IL-17 in PCS compared to the controls, but there were significant correlations between IL-17 levels and the predictive values of VC and DLCO in patients, which may indicate the involvement of IL-17 in interstitial lung injury.

IL-8 recruits and activates neutrophils to the site of infection, and increased IL-8 levels are associated with COVID-19 severity and ARDS [47]. IL-8 secretion is indirectly dependent on IL-17 [50,51].

The activation of neutrophils in COVID-19 is a negative phenomenon [6]; neutrophils in ILD are the cells of aggressive inflammatory reaction (via free radicals, metalloproteinases, and through NETosis) [15,17]. In addition, neutrophils can express PDL1 and induce the apoptosis of PD1+ T cells in COVID-19 [52].

The results of other ELISA tests, including the examination of IFNγ, TNFα, and IL-5 concentrations (no significant differences between patients and controls), are consistent with the literature [53,54,55].

In summary, PCS represents a continuation and/or regression of the inflammatory process that begins with acute SARS-COV-2 infection and may sequentially involve in its successive stages: (1) the upper respiratory tract, (2) the lung parenchyma, (3) the systemic response, and (4) the distant organs disorders [18,48,56]. In some cases, the continuation of the acute disease is represented by its chronic form, the post-COVID syndrome, which may involve organs and systems not previously affected, even in patients who have been previously vaccinated and/or had a mild form of acute infection [13,29]. It is mediated by viral antigen deposition and/or an autoimmune process [6,11,12,31]. A common site of PCS is the lower respiratory tract in the form of chronic interstitial lung disease (ILD) [3]. The prognosis is currently uncertain, but a significant proportion of patients experience spontaneous remission [27]. A subgroup of patients without remission, referred to in the study as PCS persistence, are at risk of fatal pulmonary fibrosis [24,36].

This study has several limitations. The number of participants in the study was relatively small, although the PCS groups were homogeneous and matched to the control group. However, this may have influenced the lack of statistical significance when the effect of vaccination on the occurrence of PCS was analyzed. Lung function tests of patients prior to SARS-CoV-2 infection were largely unavailable and were not analyzed. A few symptomatic patients (with PCS persistence) were excluded from the study due to systemic corticosteroid treatment. The cytoimmunological pattern of BAL may have been modified typically by comorbidities, such as bronchial asthma (increased percentage of eosinophils) and COPD (increased percentage of granulocytes) [22,57]. Low BAL fluid recovery biased the results towards a decrease in BAL cell count and lymphocyte percentage, while there was an increase in granulocyte percentage [58].

## 6. Conclusions

The immunocytological profile of BAL in post-COVID-19 (PCS) is characterized by elevated total cell counts, increased counts and percentages of lymphocytes and neutrophils, and a high BAL neutrophil:lymphocyte ratio (NLR).

A worse prognosis in PCS is associated with higher BAL NLR, a higher BAL neutrophil count and percentages, an elevated percentage of CD8+PD1+ lymphocytes, and a reduced CD4:CD8 ratio.

Th17 cells and IL-8 participate in lung PCS persistence.

## Figures and Tables

**Table 1:** Baseline data of patients with PCS. Selected lung function tests and laboratory investigations.

Variable	Patients with PCS Remission (n = 33)	Patients with PCS Persistence (n = 25)	Total PCS Patients (n = 58)	Controls (n = 11)
Age, years	55.1 ± 12.4	59.1 ± 11.1	57.7 ± 11.9	49.1 ± 16.4
Sex (M)	15 (45.5)	19 (76.0) *	34 (58.6)	6 (54.5)
Acute COVID-19 disease form				
Mild	7 (21.2)	5 (20.0)	12 (20.7)	7 (63.6)
Moderate	16 (48.5)	13 (52.0)	29 (50.0)	1 (9.1)
Severe	7 (21.2)	4 (16.0)	11 (19.0)	1 (9.1)
Critical	3 (9.1)	3 (12.0)	6 (10.3)	0
Weeks since the onset of acute COVID-19 #	33 [20–44]	25 [17–46]	28 [18–43]	38 [25–51]
Unvaccinated status	4 (12.1)	4 (16.0)	8 (13.8)	2 (18.2)
Comorbidities				
Arterial hypertension	12 (36.3)	12 (48.0)	24 (41.4)	3 (27.3)
Bronchial asthma	4 (12.1)	3 (12.0)	7 (12.1)	0
Diabetes mellitus	9 (27.2)	9 (36.0)	18 (31.0)	0
Obesity	10 (30.3)	6 (24.0)	16 (27.5)	1 (9.1)
Coronary artery disease	11 (33.3)	8 (32.0)	19 (32.8)	2 (18.2)
COPD	1 (3.3)	2 (8.0)	3 (5.2)	0
More than one comorbidity	11 (33.3)	10 (40.0)	21 (36.2)	1 (9.1)
No comorbidity	9 (27.2)	5 (20.0)	14 (24.1)	10 (90.9)
Lung function tests				
VC, predicted value%	100.9 ± 15.0	85.7 ± 15.0	97.6 ± 17.1	99.2 ± 14.5
DLCO, predicted value%	87.3 ± 12.7	58.7 ± 21.3 **	73.8 ± 24.6 *	93.3 ± 10.9
Laboratory investigations				
Blood Neutrophils, 10^3^/µL	4.3 [3.1–5.1]	3.9 [3.3–6.5]	4.2 [3.1–5.7]	4.3 [3.1–5.1]
Blood Lymphocytes, 10^3^/µL	2.0 [1.6–2.4]	1.9 [1.4–2.3]	2.0 [1.5–2.4]	2.0 [1.6–2.4]
Blood NLR	2.0 [1.6–2.8]	2.4 [1.7–3.9]	2.1 [1.6–3.6]	2.5 [1.9–4.0]
C-reactive protein, mg/L	2.1 [0.9–4.9]	3.8 [3.1–6.0] *	3.1 [1.2–6.0]	1.9 [0.6–3.9]

Data presented as numbers (percentages), mean ± SD, or median [interquartile range]. * *p* < 0.05, ** *p* < 0.01, compared to PCS remission, # on the day the BAL was performed. Abbreviations: BAL, bronchoalveolar lavage; COPD, chronic obstructive pulmonary disease; NLR, neutrophil:lymphocyte ratio; PCS, post-COVID-19 syndrome.

**Table 2:** Cytological data of BAL in PCS and controls. Major T cell subsets.

Variable	Patients with PCS Remission (n = 33)	Patients with PCS Persistence (n = 25)	Total PCS Patients (n = 58)	Controls (n = 11)
BAL fluid recovery%	47 [43–60]	46 [35–47]	47 [41–60]	47 [45–51.5]
Total cell count 10^3^/mL	270 [198–480] *	320 [165–439] *	315 [184–535] *	118 [90–167]
Macrophages%	78 [60–85]	72 [49–82] *	78 [49–85]	84.5 [81–88]
Lymphocytes%	17 [12–27] *	13.5 [10–29]	15.5 [10–27]	12 [9.5–14]
Neutrophils%	4.0 [1.9–5.9]	7.9 [4.9–16] *** #	5.1 [3.4–9.3] *	2.4 [1.1–3.2]
Eosinophils%	0.0 [0–1.1]	0.9 [0.5–1.0]	0.8 [0–1.3]	0.0 [0–0.8]
Lymphocytes 10^3^/mL	47.5 [27–81.5] **	32 [16–89.5] *	40.8 [20–89.5] *	14.4 [12.7–21]
T cells, CD3+ 10^3^/mL	44.3 [26.5–78] **	21.8 [14.5–79.7]	38.4 [17.2–79.7] *	13.1 [10.7–17.5]
Th cells, CD4+ 10^3^/mL	25.1 [14.2–55.2] *	11.7 [6.9–23.4]	19.5 [9.1–53] *	8.7 [6.7–12]
Tc cells, CD8+ 10^3^/mL	20.0 [8.2–25.2] *	10.7 [5.4–27.6] *	16.9 [7.6–27.6] *	5.1 [3.4–5.6]
NK cells, 10^3^/mL	1.5 [0.7–2.7] *	1.6 [0.7–4.2] *	1.6 [0.7–4.4] *	0.4 [0.3–0.9]

Data presented as median [interquartile range]. * *p* < 0.05, ** *p* < 0.01, *** *p* < 0.001, compared to controls; # *p* < 0.05, compared to PCS remission. Abbreviations: BAL, bronchoalveolar lavage; PCS, Post-COVID Syndrome; Th, T helper cells; Tc, T cytotoxic cells; NK, natural killer cells.

**Table 3:** Immunological data of BAL in PCS and controls. T cell immune checkpoints.

Variable	Patients with PCS Remission (n = 33)	Patients with PCS Persistence (n = 25)	Total PCS Patients (n = 58)	Controls (n = 11)
CD4+PD1+%	31.5 [5.7–68]	31.5 [16–89]	32 [5.7–89]	26 [10–52]
CD4+PDL1+%	8.1 [6.4–13]	7.0 [4.2–6.9]	7.2 [4.2–13]	5.1 [0–7.7]
CD4+CTLA4+%	8.0 [5.4–13.5]	7.2 [5.9–8.0]	7.5 [4.9–12]	8.3 [6.9–10]
CD8+PD1+%	35.5 [28–41]	43.5 [34–60.5] *	41 [28–49]	24.5 [22–44]
CD8+PDL1+%	3.9 [1.9–6.0]	2 [1.0–3.3]	2.4 [1.0–5.3]	1.9 [1.5–2.9]
CD8+CTLA4+%	7.9 [5.9–13]	6.8 [5.0–7.8]	7.1 [5.0–11]	8.1 [5.8–10]
Activated T cells CD3+HLA-DR+%	31 [24–36]	28.5 [19–39]	30.5 [20–36] *	23.5 [15–36]

Data presented as median [interquartile range]. * *p* < 0.05, compared to the controls; Abbreviations: BAL, bronchoalveolar lavage; PD1, CTLA4, cytotoxic T lymphocyte associated antygen 4; PD1, programmed death receptor 1; PDL1 (2), programmed death ligand 1 (2); PCS, Post-COVID Syndrome.

## Data Availability

The data presented in this study are openly available in RepOD at https://doi.org/10.18150/G6JD0Y (https://repod.icm.edu.pl/dataset.xhtml?persistentId=doi%3A10.18150%2FG6JD0Y&version=DRAFT).

## Supplementary Materials

The following supporting information can be downloaded at: https://www.mdpi.com/article/10.3390/jcm14103361/s1, Figure S1: Flow cytometry analysis of bronchoalveolar lavage (BAL) cells. Exemplary patients; Table S1: Murine anti-human monoclonal antibodies and murine isotype controls (manufacturer, cat. number) used in the study.

## Abbreviations

ARDS, acute respiratory distress syndrome; BAL, bronchoalveolar lavage; CD, cluster of differentiation; CCL, C-C motif chemokine ligand; COPD, chronic obstructive pulmonary disease; COVID-19, coronavirus disease 2019; CTLA4, cytotoxic T lymphocyte associated antygen 4; CXCL, C-X-C motif chemokine ligand; CXCR, C-X-C motif chemokine receptor; ELISA, enzyme-linked immunosorbent assay; HLA, human leucocyte antigen; ICP, immune checkpoints; IFN, interferon; IL, interleukin; ILD, interstitial lung disease(s); MERS, middle east respiratory syndrome; NK, natural killer; NLR, neutrophil:lymhocyte ratio; PCS, post-COVID-19 sydrome; PD1, programmed cell death protein 1; PDL1 (2), programmed cell death protein ligand 1 (2); SARS-CoV-2, severe acute respiratory syndrome-coronavirus-2; RSV, respiratory syncytial virus; TGF, transforming growth factor; TNF, tumor necrosis factor.
