# Supplementary material for: Cytoimmunological Profile of Lower Airways in Post-COVID-19 Syndrome (PCS): Predictive Value of Bronchoalveolar Lavage
Source: J Clin Med. 2025 May 12;14(10):3361. doi: 10.3390/jcm14103361 (PMC12112653; doi:10.3390/jcm14103361)

**Flow cytometry analysis of bronchoalveolar lavage (BAL) cells. Exemplary patients.**

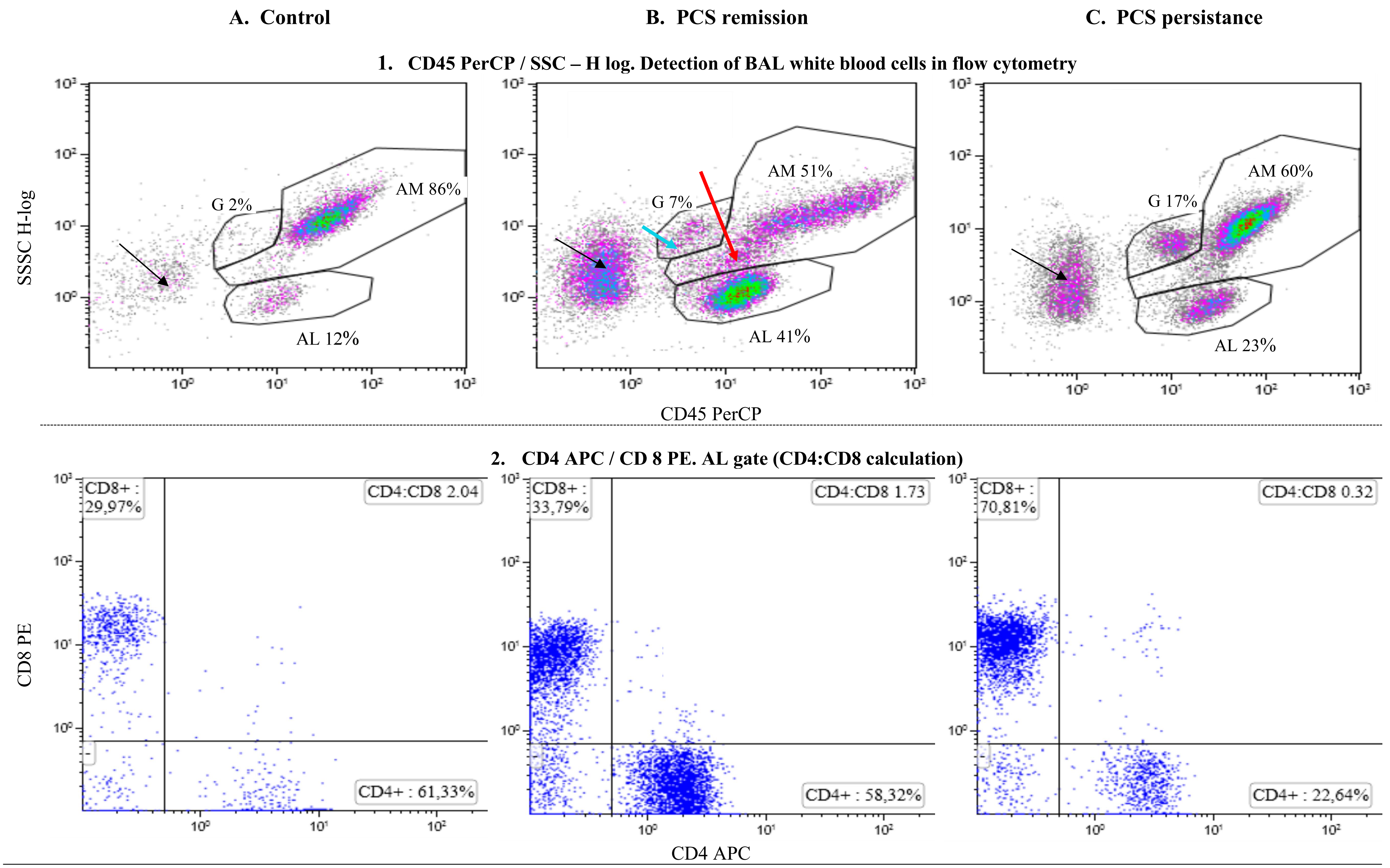

**3. CD4 APC gate. CD196 FITC / CD183 PE. Th17 vs Th1 cells**

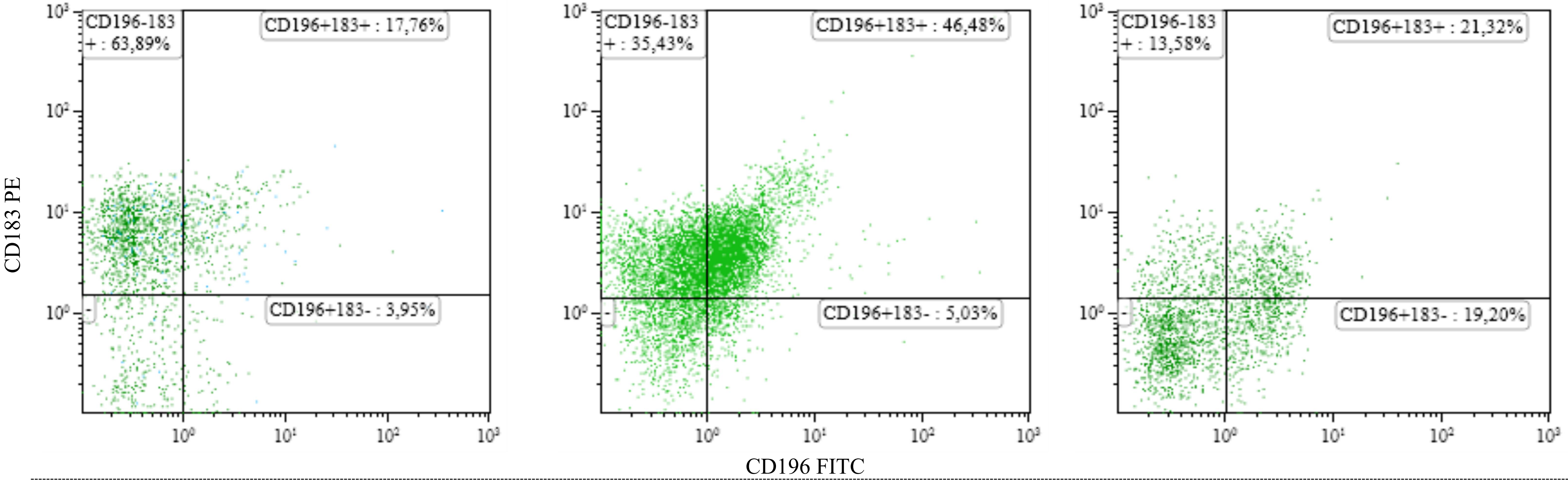


**4. CD8 APC gate. CD279 FITC / CD274 PE. T cytotoxic exhausted cells**

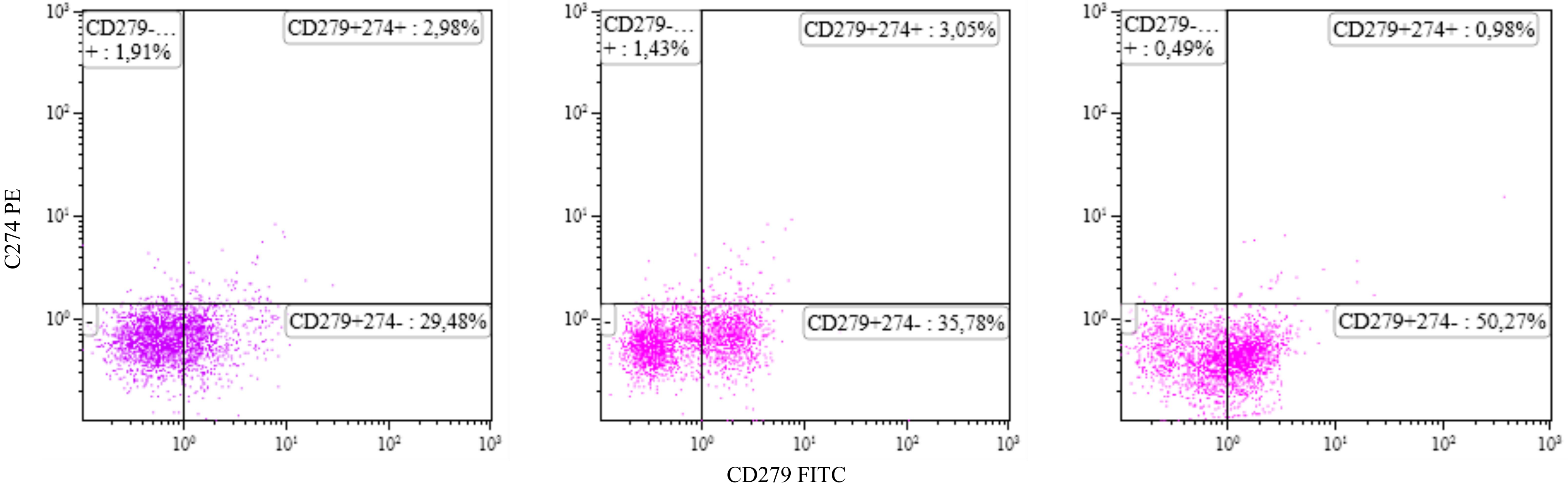

Supplement: Supplementary file 1 [file jcm-14-03361-s001.zip › jcm-3560879-Supplementary materials Figure S1.pdf]
