# Supplementary material for: Cytoimmunological Profile of Lower Airways in Post-COVID-19 Syndrome (PCS): Predictive Value of Bronchoalveolar Lavage
Source: J Clin Med. 2025 May 12;14(10):3361. doi: 10.3390/jcm14103361 (PMC12112653; doi:10.3390/jcm14103361)
Supplement: Supplementary file 1 [file jcm-14-03361-s001.zip › jcm-3560879-supplementary.pdf]

Supplementary materials.

Figure S1: **Flow cytometry analysis of bronchoalveolar lavage (BAL) cells. Exemplary patients.**

The columns show the BAL cytometric results of individual exemplary patients, i.e. from left to right: (A) control group patient, aged 45, male; (B) PCS patient with remission, 52, female; (C) patient with PCS persistence, 41, male; All are non-smokers.

The following rows show in the same patients:

(1) **CD45 PerCP / SSC - H log. Intensity plot. Detection of BAL white blood cells in flow cytometry.** CD45 (Leukocyte Common Antigen) is a sensitive and specific marker of white blood cells. In conjunction with SSC, necessarily on a logarithmic scale), it allows precise differentiation of the main BAL cell populations: alveolar macrophages (AM), alveolar lymphocytes (AL) and granulocytes (G).

Black arrows indicate debris/red blood cell contamination. The reported results of the BAL cell percentage pattern were obtained after CD45-negative debris was subtracted. Of note is the predominance of lymphocytes in patient B and the high percentage of granulocytes in patient C.

The red arrow in patient B indicates the young, monocytic forms of macrophages. The bright blue Barrow marks the disintegrating granulocytes, according to the authors' experience, neutrophils in netosis.

Please note the individual patient gating for each BAL white blood cell population.

(2) CD4 APC / CD8 PE. AL gate (CD4:CD8 calculation). Dot plot. Double positive (CD4+8+) events are treated as cell clumps and counted twice, once as CD4+ cells and once as CD8+ cells. Note the relatively high index value in the control patient and the very low value in patient C (according to our experience, the index normal value range is 0.9-4.0 [18]).

(3) CD4 APC gate. CD196 FITC / CD183 PE. Th17 vs Th1 cells. Dot plot. Th17 cells were defined as CD196+CD183- cells (right lower quadrant), Th1 cells were defined as CD196-CD183+ cells (left upper quadrant) of each dot plot. The percentages of Th17 cells as CD4+ lymphocytes are rounded 4%, 5% and 19% for patients A, B and C, respectively.

(4) CD8 APC gate. CD279 FITC / CD274 PE. T cytotoxic exhausted cells. Dot plot. Exhausted CD8+ cells were defined as PD1+ cells (CD279+CD274- or

CD279+CD274+ cells, both right lower and right upper quadrant of each dot plot). The percentages of exhausted CD8+ cells are rounded to 32%, 29% and 51% for patients A, B and C, respectively.

Abbreviations. AL, alveolar lymphocytes; AM, alveolar macrophages; APC, allophycocyanin; BAL, bronchoalveolar lavage; CD, cluster of differentiation; CTLA4, cytotoxic T lymphocyte associated antigen 4; FITC, fluorescein isothiocyanate; G, granulocytes; LCA, leukocyte common antigen; log, logarithmic scale; PCS, post-COVID-19 syndrome; PD-1, programmed death receptor 1; PE phycoerythrin; PerCP, peridinin-chlorophyll-protein.

Table S1. Murine anti-human monoclonal antibodies and murine isotype controls (manufacturer, cat. number) used in the study.

Flow cytometer fluorescence channel	**FL1**	**FL2**	**FL3**	**FL4**	**FL5**	**Notes**
**Fluorescent dye**	**FITC**	**PE**	**PerCP / PE-Cy5**	**APC**	**PE-Cy7**	
Sample 1	Isotype IgG (Biolegend 400109)	Isotype IgG (Biolegend 400111)	Isotype IgG (Biolegend 400149)	CD4, T helper cells (Biolegend 317416)	Isotype IgG (Biolegend 400126)	Control for samples 3, 6, 8
Sample 2	Isotype IgG (Biolegend 400109)	Isotype IgG (Biolegend 400111)	Isotype IgG (Biolegend 400149)	CD8, T cytotoxic cells (BD Bsc.345772)	Isotype IgG (Biolegend 400126)	Control for samples 3, 7
Sample3	Isotype IgG (Biolegend 400109)	Isotype IgG (Biolegend 400111)	CD45, LCA (BD Bsc345809)	Isotype IgG (Biolegend 401209)		Detection of non-lymphocyte cells in BAL lymphocyte gate
Sample4	CD3. T cells	CD8, Tcytotoxic cells	CD45, LCA	CD4, T helper cells		MULTI 1 set (BD Biosciences 342417)
Sample 5	CD3, T cells	CD 16+56, NKcells	CD45,LCA	CD19, B cells		MULTI 2 set (BD Biosciences 342416)
Sample6	CD279, PD1 (Biolegend	CD274, PD-L1 (Biolegend	CD152, CTLA4 (Biolegend	CD4, T helper (Biolegend	CD273, PD-L2 (BD	Detection of immune checkpoints on Th cells

	329904)	393608)	369644)	317416)	Bsc345512)	
Sample7	CD279, PD1 (Biolegend 329904)	CD274, PD-L1 (Biolegend 393608)	CD152, CTLA4 (Biolegend 369644)	CD8, T cytotoxic (BD Bsc345772)	CD273, PD-L2 (BD Bsc345512)	Detection of immune checkpoints on Tc cells
Sample8	CD196, CCR6 (Biolegend 353412)	CD183, CXCR3 (Biolegend 353706)	CD194, CCR4 (Biolegend 359406)	CD4, T helper (Biolegend 317416)		Th1/Th2/Th17 polarization
Sample9	Unstained sample					

APC, allophycocyanin; BD, Becton-Dickinson; BD Bsc, Becton-Dickinson Biosciences; CCR4, C-C motifchemokine receptor 4; CCR6, C-C motifchemokine receptor 6; CTLA4, cytotoxic T lymphocyte associated antigen 4; CXCR3, C-X-C motifchemokine receptor 3;Cy5.5, Cyanine 5.5.; FITC, fluorescein isothiocyanate; FL, fluorescence; LCA, leukocyte common antigen; PD1, programmed death receptor 1;PD-L1 (2), programmed death ligand 1 (2);PE Cy 5.5, phycoerythrin cyanine 5.5; PerCP, peridinin-chlorophyll-protein
